# Characterization of Social Isolation and Perception of Loneliness in a Group of Chilean Older People post‐pandemic

**DOI:** 10.1002/alz.089300

**Published:** 2025-01-09

**Authors:** Christine Gierke, Carlos F. Navarro, Melissa Martinez, Carolina Delgado, Paul H Delano, Vicente Medel, Alexis Leiva, Cristina De Gatica, Victor Vidal, Simon San Martin, Ximena Garcia, Mauricio Cerda, Rodrigo C. Vergara, Gonzalo Farías

**Affiliations:** ^1^ Universidad de Chile, Santiago Chile; ^2^ Hospital Clínico Universidad de Chile, Santiago Chile; ^3^ Biomedical Neuroscience Institute (BNI), ICBM, Faculty of Medicine, University of Chile, Chile, Santiago Chile; ^4^ Universidad de Chile, Santiago, Region Metropolitana Chile; ^5^ Hospital Clínico Universidad de Chile, Santiago, Region Metropolitana Chile; ^6^ Latin American Brain Health Institute (BrainLat), Universidad Adolfo Ibáñez, Santiago, Región Metropolitana Chile; ^7^ Biomedical Neuroscience Institute (BNI), ICBM, Faculty of Medicine, University of Chile, Santiago Chile; ^8^ Facultad de Psicología y Humanidades, Universidad San Sebastián, Valdivia Chile; ^9^ Centro Nacional de Inteligencia Artificial CENIA, Santiago Chile; ^10^ Universidad de Chile., Santiago Chile

## Abstract

**Background:**

During the pandemic, Social Isolation (SI) and the perception of loneliness emerged as critical factors associated with significant psychological and physical impacts (Tyrrell, C. & Williams, N., 2020; González, D., 2021).

This study examines gender differences and explores the impact of social isolation and the perception of loneliness on self‐sufficient older people in Santiago, Chile, in the post‐pandemic period (March to November 2022). The focus is on understanding the relationship between these factors and psychiatric symptoms, utilizing specific scales to evaluate these variables.

The pandemic introduced three main variables that affected older individuals: the severity of the disease and the fear of contagion; the effects of social isolation, and the perception of vulnerability, which was amplified by the media (NCHS, 2020; CEVE‐UC, 2022; Smith, B. & Lim, M., 2020; Ayalon et al., 2020; Monahan et al., 2020; Vervaeke & Meisner, 2020).

**Method:**

A descriptive cross‐sectional study included 150 participants (ages 60‐87). Surveys administered were the Steptoe Social Isolation Index, Three‐Item UCLA Loneliness Scale, Yesavage Geriatric Depression Scale (GDS‐15), current concern about the pandemic (Likert Scale, 1‐10), and gender differences in these variables. JAMOVI software version 2.36 was utilized for data processing and statistical analysis.

**Result:**

From March to December 2022, 150 older adults were surveyed (Mean = 69.5, Median = 69, SD = 5.93, Variance = 35.11). The sample comprised 68% women and 32% men (Women = 102, Men = 48). Of the variables analyzed, 42% of the sample experienced Social Isolation (Chi‐square = 3.2, p = 0.696 / U = 2395.0, p = 0.825). 26% perceived Loneliness (Chi‐square = 6.10, p = 0.412 / U = 2210.0, p = 0.290), 30% indicated depression (Chi‐square = 7.94, p = 0.848 / U = 2270.0, p = 0.470), and 91% expressed concern about the pandemic (Chi‐square = 0.10, p = 1 / U = 2442, p = 0.980).

**Conclusion:**

The results suggest that, although there are no significant gender differences in individual variables, there are significant correlations between all combinations of the variables (Social Isolation, Loneliness, GDS‐15, and Pandemic Concern). Among men, significant correlations were found between Social Isolation and Loneliness (rho = 0.432, p = 0.002), Loneliness and GDS‐15 (rho = 0.299, p = 0.03), and Loneliness and Age (rho = 0.381, p = 0.008). In women, all correlations were significant, except with age, between Social Isolation and Loneliness (rho = 0.419, p≤0.001), Social Isolation and GDS‐15 (rho = 0.279, p = 0.005), and Loneliness and GDS‐15 (rho = 0.474, p≤0.001).

